# Enzyme inhibitory and antioxidant activities of traditional medicinal plants: Potential application in the management of hyperglycemia

**DOI:** 10.1186/1472-6882-12-77

**Published:** 2012-06-19

**Authors:** Vandana Gulati, Ian H Harding, Enzo A Palombo

**Affiliations:** 1 Environment and Biotechnology Centre, Faculty of Life and Social Sciences, Swinburne University of Technology, PO Box 218, Hawthorn 3122 VIC, Australia

**Keywords:** Anti-diabetic, Enzyme inhibition, Antioxidant

## Abstract

**Background:**

Traditional Indian and Australian medicinal plant extracts were investigated to determine their therapeutic potential to inhibit key enzymes in carbohydrate metabolism, which has relevance to the management of hyperglycemia and type 2 diabetes. The antioxidant activities were also assessed.

**Methods:**

The evaluation of enzyme inhibitory activity of seven Australian aboriginal medicinal plants and five Indian Ayurvedic plants was carried out against α-amylase and α-glucosidase. Antioxidant activity was determined by measuring (i) the scavenging effect of plant extracts against 2, 2-diphenyl-1-picryl hydrazyl (DPPH) and 2, 2′-azinobis-3-ethylbenzothiazoline-6-sulfonate (ABTS) and (ii) ferric reducing power. Total phenolic and total flavonoid contents were also determined.

**Results:**

Of the twelve plant extracts evaluated, the highest inhibitory activity against both α-amylase and α-glucosidase enzymes was exerted by *Santalum spicatum* and *Pterocarpus marsupium* with IC_50_ values of 5.43 μg/ml and 0.9 μg/ml, respectively, and 5.16 μg/ml and 1.06 μg/ml, respectively. However, the extracts of *Acacia ligulata* (IC_50_ = 1.01 μg/ml), *Beyeria leshnaultii* (0.39 μg/ml), *Mucuna pruriens* (0.8 μg/ml) and *Boerhaavia diffusa* (1.72 μg/ml) exhibited considerable activity against α-glucosidase enzyme only. The free radical scavenging activity was found to be prominent in extracts of *Acacia kempeana*, *Acacia ligulata* followed by *Euphorbia drummondii* against both DPPH and ABTS. The reducing power was more pronounced in *Euphorbia drummondii* and *Pterocarpus marsupium* extracts. The phenolic and flavonoid contents ranged from 0.42 to 30.27 μg/mg equivalent of gallic acid and 0.51 to 32.94 μg/mg equivalent of quercetin, respectively, in all plant extracts. Pearson’s correlation coefficient between total flavonoids and total phenolics was 0.796.

**Conclusion:**

The results obtained in this study showed that most of the plant extracts have good potential for the management of hyperglycemia, diabetes and the related condition of oxidative stress.

## Background

Diabetes mellitus is an important metabolic syndrome. The increasing worldwide incidence of diabetes mellitus in adults constitutes a global public health burden. The World Health Organization (WHO) estimates that currently more than 180 million people worldwide have diabetes and it is likely to double by 2030, with India, China and United States predicted to have the largest number of affected individuals [1,2]. Many plants and their active chemical compounds have demonstrated activity in the treatment of various disorders [3]. According to ethnobotanical information, more than 800 plants are used as traditional remedies in one or other form for the treatment of diabetes [4]. The management of diabetes without any side effects is still a challenge; therefore plants continue to play an important role in the discovery of new compounds for the treatment of this disease.

The management of diabetes can be achieved by reducing post-prandial hyperglycemia by delaying the activities of the enzymes α-amylase and α-glucosidase which are responsible for the digestion of carbohydrates and absorption of glucose in the digestive tract, respectively [5,6]. Drugs derived from natural products have played a major role in the development of pharmaceutical treatments for diabetes. Metformin, the single most prescribed agent for the treatment of diabetes, originated from herbal medicine [7,8]. A plant-derived antidiabetic agent, galegine, was isolated from *Galega officinalis*. Experimental and clinical evaluations provided the pharmacological and chemical basis for the subsequent discovery of metformin [7,9]. 1- deoxynojirimycin (DNJ), a potent α-glucosidase Inhibitor, was isolated from the water extract of leaves of the mulberry tree (*Morus alba* L.) [10].

There are many cellular biochemical pathways and environmental toxins which produce reactive oxygen species (ROS) [11] and contribute to the development of diseases such as cancer, cardiovascular disorders, diabetes, cataracts and many neurodegenerative diseases [12]. Many studies have confirmed that plants and foods rich in polyphenolic content are effective scavengers of free radicals, thus helping in the prevention of these diseases through their antioxidant activity [13]. Antioxidants which are present in plants, herbs and dietary sources help in preventing vascular diseases in diabetic patients [14]. Tannins and flavonoids are the secondary metabolites in plants considered to be the natural source of antioxidants which prevent destruction of β-cells and diabetes-induced ROS formation [15]. Thus, it is a good strategy to manage diabetes as a whole with plants which show good enzyme inhibitory and antioxidant activities [16]. Therefore, the aim of our study was to screen some traditional Australian aboriginal plants and Ayurvedic Indian plant extracts to determine those which showed promising enzyme inhibitory and antioxidant activities.

## Methods

### Plants

The Australian aboriginal plants were selected on the basis of availability and their known medicinal activities. The Indian Ayurvedic plants were selected according to their reported anti-diabetic potential. These plants were known to possess anti-diabetic action and but not all plants had been screened using enzymatic inhibition assays used in this study. Seven Australian aboriginal medicinal plant extracts were obtained from the University of South Australia, Adelaide, Australia. Powdered extracts of five Indian Ayurvedic plants were provided by Promed Research Centre, Gurgaon, India. Table 1 shows the ethnobotanical uses of the plants used in this study. Many of the plants screened here have been used as food or food supplements, suggesting that they are safe to take orally. Seeds and gums of *Acacia* species are edible and, as this plant grows in harsh environments, it is commonly known as “dead finish”, *Santalum lanceolatum* (SL) has sweet fruits which are eaten fresh and the decoction of the inner bark of *S. spicatum* (SS) was drunk to get relief from coughs [17]. Fruits of *Eugenia jambolana* (EJ), called blackberries in English, are eaten fresh, are rich in polyphenols, are widely distributed in India and are known to reduce glucose [18]. Seeds of *Mucuna pruriens* (MP), also known as velvet beans, are cooked or can be eaten raw [19] and in Central America the roasted and ground seeds are used as a substitute for coffee [20]. Tuberous roots of *Curculigo orchioides* (CO) are eaten to maintain vitality, strength and have aphrodisiac effects [21]. Tribal people of West Bengal eat *Boerrhaavia diffusa* (BD) as a vegetable, while in the Assam state of India, this plant is also cooked and eaten [22].

**Table 1:** Traditional uses of Australian aboriginal and Indian Ayurvedic plants used in this study

** Plant**	** Family**	** Use**	**References**
***Acacia kempeana*F. Muell.**	Mimosaceae	Chest infection, severe cold, general sickness	[23,24]
***Acacia*.*tetragonophylla*F. Muell.**	Mimosaceae	Cough, treatment of circumcision wounds, dysentery	[25]
***Acacia ligulata*Cunn. ex Benth.**	Mimosaceae	Cough, cold, chest infection, general illness	[24,26,27]
***Beyeria lechenaultii*(DC.) Baillon**	Euphorbiaceae	General sickness, fever	[26]
***Euphorbia drummondii*Boiss.**	Euphorbiaceae	Skin sores, genital sores, fever, dysentery	[23]
***Santalum lanceolatum*R. Br.**	Santalaceae	Cold, malaise, sore throat, venereal diseases, painful urination	[23]
***Santalum spicatum*(R. Br.) A. DC.**	Santalaceae	Cough	[26]
***Boerhaavia diffusa*Linn.**	Nyctaginaceae	Diuretic, anti-inflammatory, antifibrinolytic, anticonvulsant, antibacterial, antihepatotoxic, antidiabetic	[22,28]
***Curculigo orchioides*Gaertn.**	Amaryllidaceae	Demulscent, diuretic, aphrodisiac, asthma, jaundice, hepatoprotective	[21,29]
***Eugenia jambolana*Lam.**	Myrtaceae	Bronchitis, asthma, sore throat, diabetes, dysentery, antibacterial, antioxidant	[18,30,31]
***Mucuna Pruriens*Linn.**	Leguminoseae	Anti-parkinson, hypoglycemic, hypo-cholestrolemic, antioxidant, antitumour, antimicrobial	[32,33]
***Pterocarpus marsupium*Roxb.**	Fabaceae	Antidiabetic, anticataract, cardiotonic, hepatoprotective	[32,34]

### Preparation of extracts

The preparation of Australian aboriginal plants extracts and information about voucher specimens have previously been described [23]. The Indian Ayurvedic plants were provided as dried powders by Promed Research Centre, India, with the following batch codes: PROM/PTMA-01 (PM), PROM/BD-43 (BD), PROM/CUOR-15 (CO), PROM/EUJA-10 (EJ) and PROM/MUPR-05 (MP). Five grams of powder were soaked overnight in 50 ml ethanol and filtered with Whatmann filter paper No. 1. The filtrates were concentrated *in vacuo* in a rotary evaporator at 55°C and reconstituted in ethanol at 250 μg/μl as a stock solution which was used to make working solutions of various concentrations. Enzymes and chemicals (α-amylase enzyme from porcine pancreas, yeast α-glucosidase, acarbose, *p*-nitro phenyl glucopyranoside (p-NPG) and 1,1-diphenyl-2-picrylhydrazyl radical (DPPH), 2,2′-azinobis-3-ethylbenzothiazoline-6-sulfonate (ABTS), butylated hydroxyl toluene (BHT), potassium ferricyanide, trichloroacetic acid, ascorbic acid, ferric chloride, Folin-Ciocalteu reagent, quercetin, gallic acid and aluminium chloride were purchased from Sigma-Aldrich, Australia.

### Amylase inhibition screening assay

The α-amylase inhibitory assay was modified from Correia et al. [35]. Twenty μl of porcine pancreatic α-amylase solution (EC 3.2.1.1; equivalent to 3000 U in 50 mM phosphate buffer, pH 6.9) were mixed with 15 μl of plant extract and incubated at 37°C for 45 minutes. After incubation, the plant - enzyme mixture was applied to a sterile paper disc and placed onto the center of petri plates containing medium consisting of 1% (w/v) agar and 1% (w/v) starch in distilled water. Plates were allowed to stand for 3 days at 25°C then stained with iodine and allowed to stand for 15 min. The diameter of the clear zone was measured and used to calculate the amylase inhibitory activity. As a control, the enzyme was mixed with the solvent in which the plants were extracted (ethanol) and applied onto the sterile disc. Results were expressed as percentage (%) amylase inhibition = {(diameter of control – diameter of sample)/diameter of the control} × 100.

### Amylase inhibition assay by quantitative starch hydrolysis

The α-amylase inhibitory activity was determined [36] using porcine pancreatic α-amylase solution (EC 3.2.1.1) type VI B. To 125 μl of different plant extract concentrations (range 1.56 μg/ml to 500 μg/ml), α-amylase solution (0.5 mg/ml in 0.02 M sodium phosphate buffer) was mixed and the reaction mixture was pre-incubated for 10 minutes at room temperature. After pre-incubation, 25 μl of 1% (w/v) starch solution were added every 5 seconds for a total of 125 μl. The reaction mixture was again incubated for 10 minutes at room temperature. The reaction was terminated by adding 250 μl of 3, 5-dinitro salicylic acid reagent. The tubes were placed in boiling water bath for 5 minutes and then cooled at room temperature. The reaction mixture was diluted by adding 5000 μl of distilled water. The generation of maltose was quantified by measuring the absorbance at 540 nm of 3-amino-5-nitrosalicylic acid (from reduction of 3, 5-dinitrosalicylic acid [37]) using a UV-visible spectrophotometer. The control was buffer treated in the same way as plant samples. The standard used was acarbose (concentration range 1.56 μg/ml to 500 μg/ml). Results were expressed as percentage (%) amylase inhibition = {(absorbance of control_540nm_ – absorbanceof samples_540nm_)/absorbance of the control_540nm_} × 100.

### Glucosidase inhibition assay

The α-glucosidase inhibition assay was modified from [Apostolidis et al. 38] using yeast α-glucosidase (EC – 2328898). A volume of 25 μl of plant extract (range 0.35 μg/ml to 100 μg/ml) was mixed with 50 μl of α-glucosidase enzyme (0.1 U/ml in 0.1 M potassium phosphate buffer solution, pH 6.9) in 96 well plates and incubated at 37°C for 30 minutes. After pre-incubation, 25 μl of 5 mM pNPG in 0.1 M phosphate buffer were added to each well and the reaction mixture was incubated again at 37°C for 30 minutes. Thirty μl of 0.1 M sodium carbonate solution were added to the above reaction mixture and incubated again for 20 minutes at 37°C. Before and after incubation, the absorbance was measured at 405 nm and compared to the control that contained 25 μl of buffer solution instead of plant extract. The standard used was acarbose (concentration range 0.35 μg/ml to 100 μg/ml). The α-glucosidase activity was determined by measuring release of p-nitrophenol from p-nitrophenyl α-D-glucopyranoside [39]. The α-glucosidase inhibitory activity was expressed as

$$\begin{array}{c} \;Percentage\left(\%\right)inhibition\\ =\{(absorbance\;of\;control_{405nm}\\ \;-absorbance\;of\;samples_{405nm})\\ \;/absorbance\;of\;the\;control{\;}_{405nm}\}\times 100 \end{array}$$

### Total phenolic content assay

The total phenolic content was quantified using a modified version of the assay described by [Singleton et al. 40] using Folin-Ciocalteu reagent. Twenty μl of plant sample or gallic acid (standard phenolic compound) were diluted with 1580 μl of distilled water and then mixed with 100 μl of 2 N Folin-Ciocalteu reagent. The mixture was shaken and kept for 6 minutes, after which 300 μl of 5% aqueous Na_2_CO_3_ solution were added and mixed properly. The mixture was incubated for 2 hours at 20°C. The absorbance was measured for all the samples at 765 nm. A standard curve was prepared using 25–1000 mg/l of gallic acid. The total phenolic values were expressed in terms of gallic acid equivalents (μg/mg of dry mass). The blank (distilled water) was treated in the same way as the samples and the dilution factor was taken into account for the samples where dilution was performed.

### Determination of total flavonoids

The total flavonoids were determined using a modification of the assay described by [Dixit et al. 41]. Plant samples (250 μl) were diluted with 1250 μl of distilled water and 75 μl of 5% NaNO_2_ were added to the sample together with 150 μl of 10% aluminum chloride. After mixing and incubation for 5 minutes, 500 μl of 1 M NaOH were added to the reaction mixture and a total volume of 2500 μl was made up with distilled water. Following vigorous mixing, the absorbance was measured at 510 nm. A standard curve was prepared using 25–1000 mg/l of quercetin. Results were expressed as μg of quercetin equivalents per milligram of dry mass of the plant extract.

### Antioxidant activity determined by 1, 1-diphenyl-2-picrylhydrazyl (DPPH) radical inhibition

The DPPH scavenging activity was determined by an assay modified from [Kwon et al. 42]. To 150 μl of 0.1 mM DPPH in methanol, a volume of 50 μl of plant extract (range 20 to 1000 μg/ml) was mixed and kept in the dark at room temperature for 60 minutes. After incubation, the absorbance was recorded at 490 nm. The results were compared with the control which contained 50 μl of ethanol instead of plant extracts. The positive controls were butyl hydroxyl toluene (BHT) and ascorbic acid in concentration range 3.12 to 250 μg/ml. The antioxidant activity was expressed aspercentage (%) inhibition = {(absorbance of control_490nm_ – absorbance of samples_490nm_)/ absorbance of the control_490nm_} × 100.

### Antioxidant activity determined by 2,2′-azinobis-3-ethylbenzothiazoline-6-sulfonate (ABTS) radical inhibition

The free radical scavenging activity of plant extracts was also studied using the ABTS radical cation. The decolorization assay is based on reduction of the ABTS +  · radical by plant extracts having antioxidant capacity [43]. ABTS radical was dissolved in deionized water to make a 7 mM solution and 2.45 mM potassium persulfate solution was added to the same. To produce the ABTS free radical cation, the mixture was allowed to stand in dark at room temperature for 12 to 16 hours. The free radical solution of ABTS was diluted with ethanol to an absorbance of 0.7 at 734 nm for the assay. To 990 μl of ABTS free radical solution, a volume of 10 μl of plant extract (range 1 to 1000 μg/ml) was mixed and kept in the dark at room temperature for 60 minutes. After incubation, the absorbance was recorded at 734 nm. The results were compared with the control which contained 10 μl of ethanol instead of plant extracts. The positive controls were BHT and ascorbic acid in concentration range 1.56 to 250 μg/ml. The antioxidant activity was expressed as percentage (%) inhibition = {(absorbance of control_734nm_ – absorbance of samples_734nm_)/ absorbance of the control_734nm_} × 100.

### Ferric reducing power assay

The ferric reducing power assay was carried out as described by [Fawole et al. 44] using 30 μl plant extracts and standards (BHT and ascorbic acid) of different concentrations (range 25 to 1000 μg/ml) added to 96 well plates along with 40 μl of 0.2 M potassium phosphate buffer (pH 7.2) and 40 μl of potassium ferricyanide (1% w/v). The reaction mixtures were incubated at 50°C for 20 minutes. After incubation, 40 μl of trichloroacetic acid (10% w/v), 150 μl distilled water and 30 μl of ferric chloride (0.1% w/v) were added and the reaction mixture again incubated for 30 minutes at room temperature in the dark. Absorbance was recorded at 630 nm using a microplate reader and the positive controls were BHT and ascorbic acid whereas the negative control was buffer. The absorbance of each sample was plotted against concentration and compared with the standards.

### Statistical analysis

All samples were analyzed in triplicate. Data are presented as mean ± standard error mean (SEM). Differences were evaluated by one-way analysis of variance (ANOVA) test completed by a Bonferroni’s multicomparison test. Differences were considered significant at p < 0.01. The concentration giving 50% inhibition (IC_50_) was calculated by non-linear regression with the use of Graphpad Prism Version 5.0 for Windows (GraphPad Software, San Diego, CA, USA) ( http://www.graphpad.com). The dose–response curve was obtained by plotting the percentage inhibition versus concentration [37].

## Results and discussion

The inhibition of digestive enzymes, such as α-amylase and α-glucosidase, has been considered to be an effective strategy to control blood glucose. Agents based on natural products are particularly attractive as side effects are minimal and the therapies are well-tolerated compared to the other oral hypoglycemic agents currently available [5,36]. The present study was therefore designed to investigate the bioactive properties of twelve traditional medicinal plants relevant to the management of hyperglycemia and type 2 diabetes. These properties included inhibition of α-amylase and α-glucosidase enzymes and antioxidant potential. The total phenolic and total flavonoid contents of the extracts were also determined.

Type 2 diabetes is a global health challenge and the WHO has recommended research and use of complementary medicines for the management of this disease. Type 2 diabetes was previously considered as maturity-onset diabetes but, due to increasing rates of obesity, there is an increasing risk of developing this disease in childhood [45,46]. The goal of treatment is to maintain normal levels of blood glucose and prevent the development of skin infections, diabetic nephropathy and cardiovascular disorders [47].

The results of preliminary agar diffusion amylase inhibition assays indicated that all of the Australian aboriginal plant extracts showed complete inhibition of α-amylase enzyme such that no hydrolysis of starch was evident. Among the Indian Ayurvedic plant extracts, only *Eugenia jambolana and Curculigo orchioides* showed complete inhibition at 250 mg/ml. *Mucuna pruriens*, *Boerhaavia diffusa* and *Pterocarpus marsupium* extracts showed partial inhibition. While the majority of the extracts demonstrated potent α-amylase inhibiting activity, some of the Australian plant extracts were particularly active ( *Acacia ligulata* and *Acacia tetragonophylla*) and showed activity at concentrations lower than those of the other extracts.

**Figure 1:**
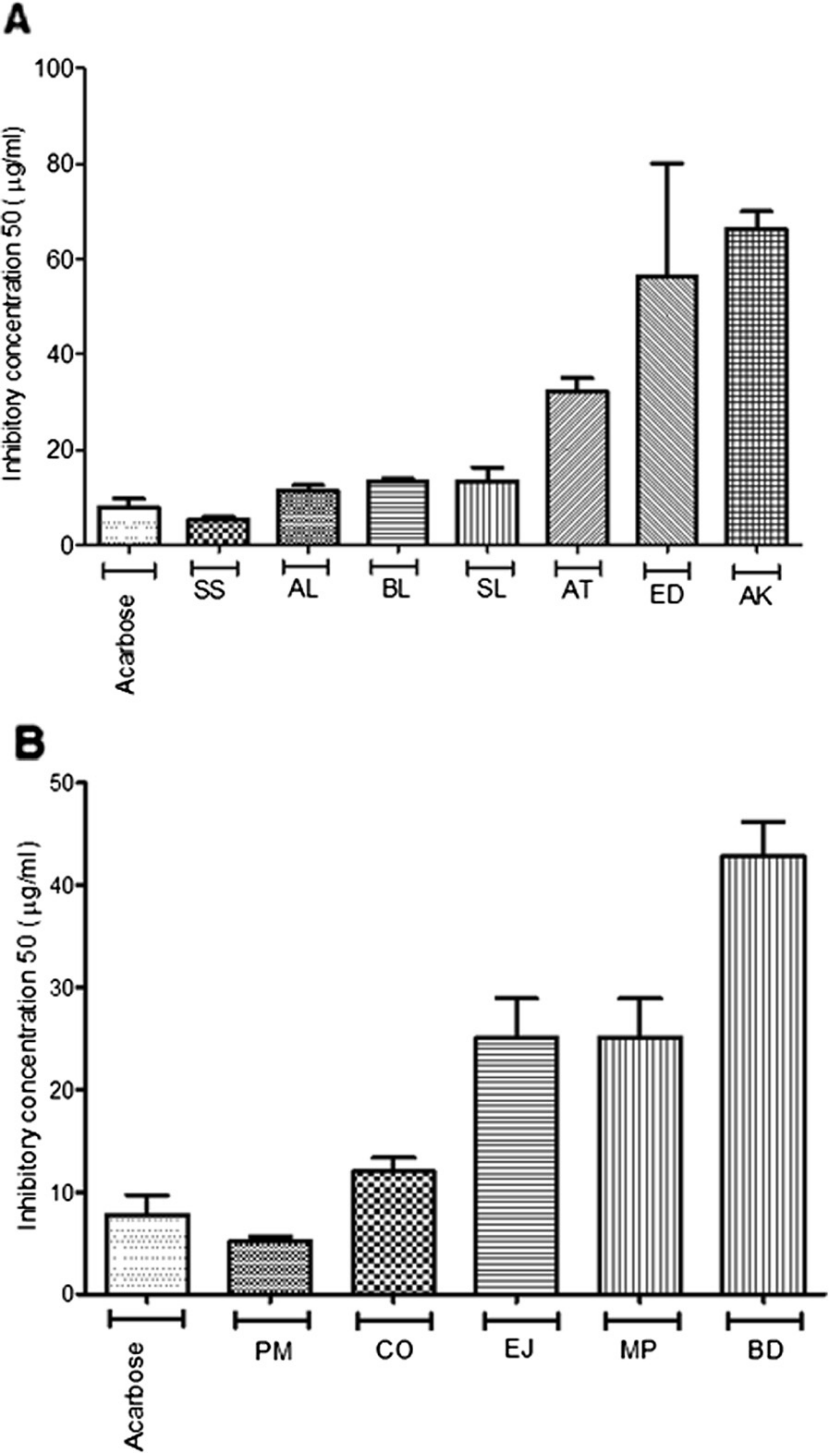
**α-amylase inhibition of selected plants.** α-amylase IC_50_ of ethanolic extracts of (**A**) Australian aboriginal plants and ( **B**) Indian Ayurvedic plants. Each value represents the mean ± SEM of triplicates experiments.

**Figure 2:**
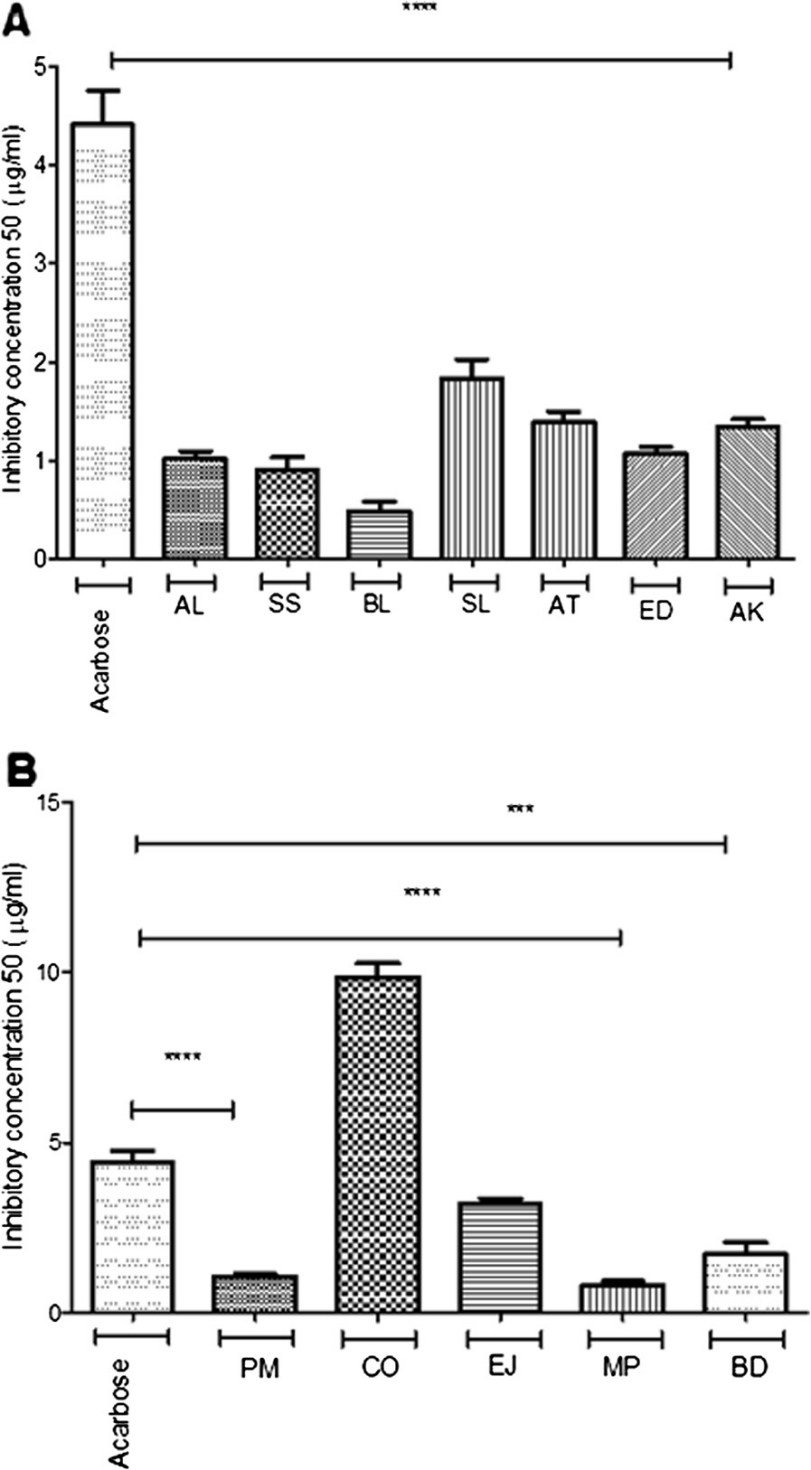
**α-glucosidase inhibition of selected plants.** α-glucosidase IC_50_ of ethanolic extracts of (**A**) Australian aboriginal plants and ( **B**) Indian Ayurvedic plants. Each value represents the mean ± SEM of triplicates experiments. **** p < 0.001 and *** p < 0.01 indicate a significant difference between acarbose and plant extracts, one-way ANOVA post-hoc Bonferroni's multiple comparison test.

In the amylase assay (Figure 1A), the positive control acarbose showed an IC_50_ of 7.81 μg/ml and the Australian plant extracts which exerted higher amylase inhibitory activity were SS (5.53 μg/ml), AL, BL and SL. We did not observe similar activity with extracts of *Acacia tetragonophylla* (AT) *, Euphorbia drumondii* (ED) and *Acacia kempeana* (AK) which showed IC_50_ values in the range 32 to 66 μg/ml. The same plant extracts showed statistically significant low IC_50_ (in the range 0.48 to 1.83 μg/ml) as compared to acarbose (4.41 μg/ml) for glucosidase inhibition (Figure 2A).

For amylase inhibition, among the five Indian plant extracts screened, only PM extract showed a lower, but not statistically significant, IC_50_ of 5.16 μg/ml compared to acarbose (Figure 1B). EJ, CO and MP extracts showed IC_50_ in the range 12.01 to 42.81 μg/ml. Only MP and PM extracts showed statistically significant lower IC_50_ value for glucosidase inhibition as compared to acarbose (Figure 2B).

DPPH is a radical, purple in colour, which is reduced to the yellow coloured diphenylpicrylhydrazine by plant extracts. This antioxidant assay is based on the reduction of alcoholic DPPH solution in the presence of hydrogen-donating antioxidant due to the formation of the non-radical form, DPPH-H [48].

**Figure 3:**
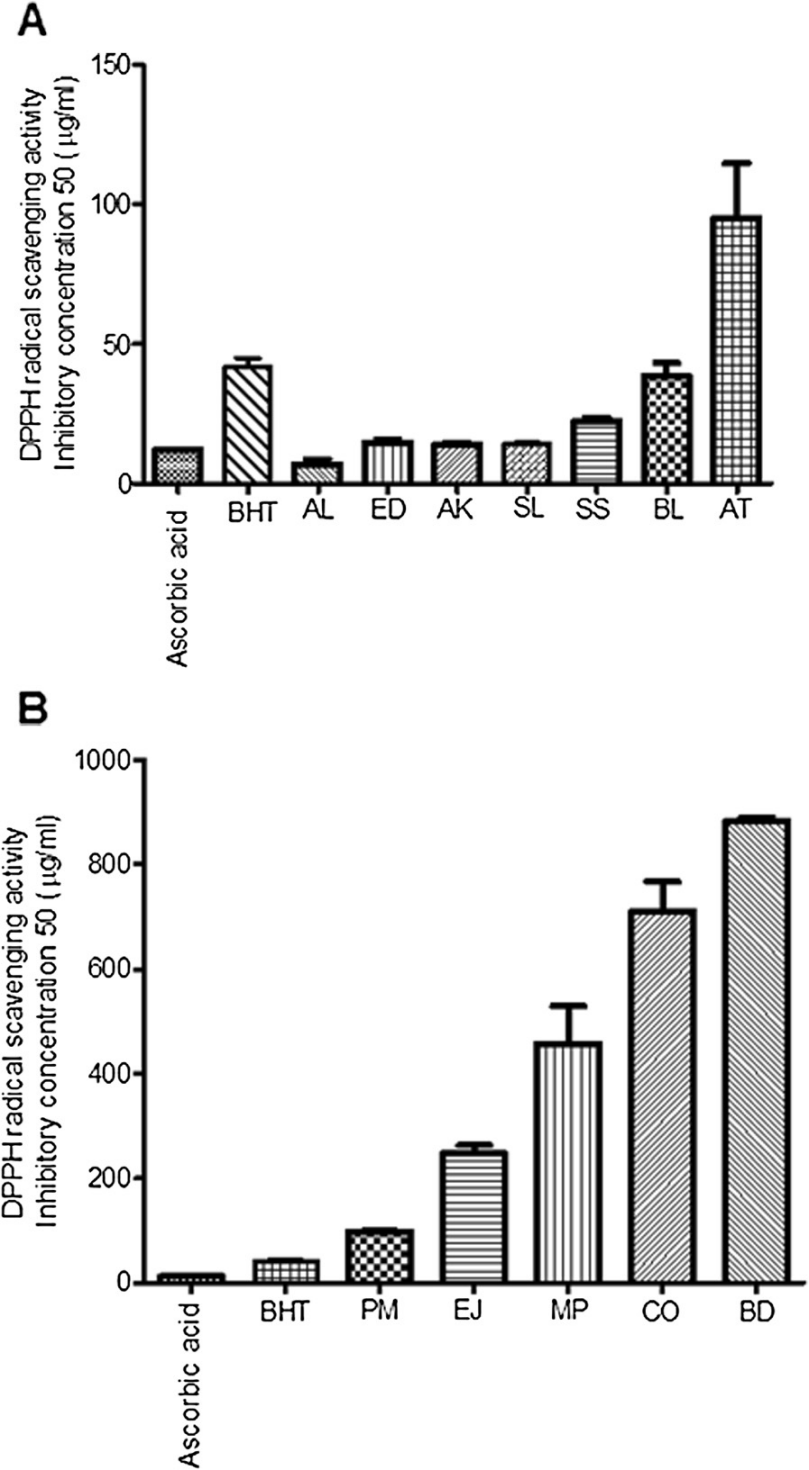
**DPPH radical scavenging activity of selected plants.** DPPH radical scavenging IC_50_ of ethanolic extracts of (**A**) Australian aboriginal plants and ( **B**) Indian Ayurvedic plants. Each value represents the mean ± SEM of triplicates experiments.

The positive controls, ascorbic acid and BHT, were used for DPPH, ABTS and ferric reducing power assays. IC_50_ values (Figure 3A and 3B) were calculated for all the extracts and controls and were found to be 12.37 and 41.82 μg/ml for ascorbic acid and BHT, respectively. Among the Australian plant extracts, AL showed nearly half the IC_50_ value of 6.98 μg/ml whereas the rest of the plant extracts exerted similar IC_50_ to that of ascorbic acid with AT having a five-fold higher IC_50_ value. The Indian plant extracts exerted IC_50_ in range 97 to 883 μg/ml.

**Figure 4:**
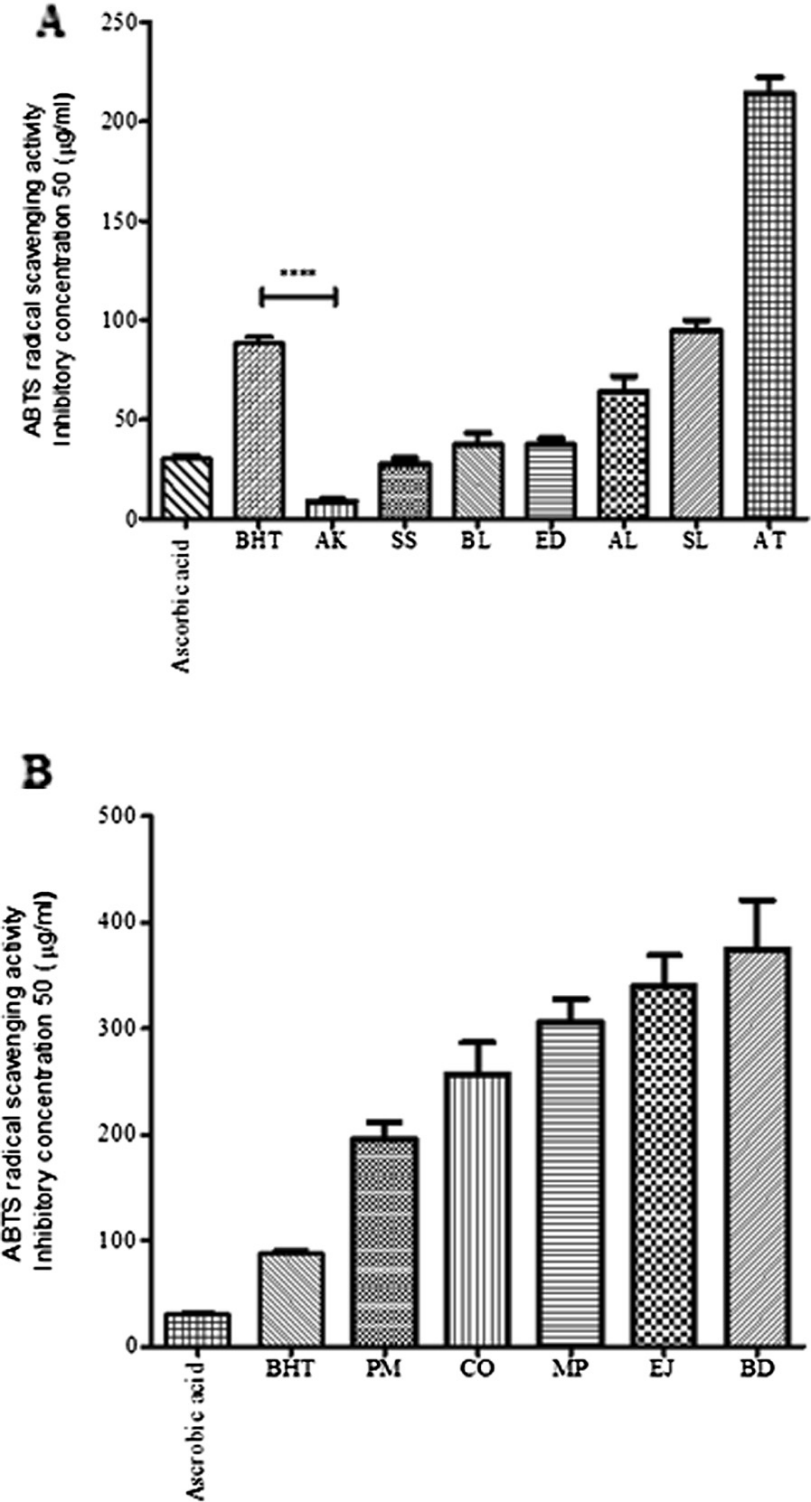
**ABTS radical scavenging activity of selected plants.** ABTS radical scavenging IC_50_ of ethanolic extracts of (**A**) Australian aboriginal plants and ( **B**) Indian Ayurvedic plants. Each value represents the mean ± SEM of triplicates experiments. **** p < 0.001 indicates significant difference between BHT and plant extract, one-way ANOVA post-hoc Bonferroni's multiple comparison test.

ABTS is a stable free radical, bluish-green in colour and the antioxidant assay is based on the reduction of ABTS solution by plant extracts. IC_50_ values (Figure 4A and 4B) for positive controls, ascorbic acid and BHT, were found to be 30.20 and 88.24 μg/ml, respectively. Out of seven Australian plant extracts screened for ABTS radical scavenging activity, AK showed significantly lower IC_50_ as compared to BHT. Barring SL and AT, the rest of the plant extracts tested showed IC_50_ value equal or less than the positive controls tested. The five Indian plant extracts tested failed to show any significant activity as compared to positive controls and the IC_50_ value was in the range from 195.96 to 374.70 μg/ml.

**Figure 5:**
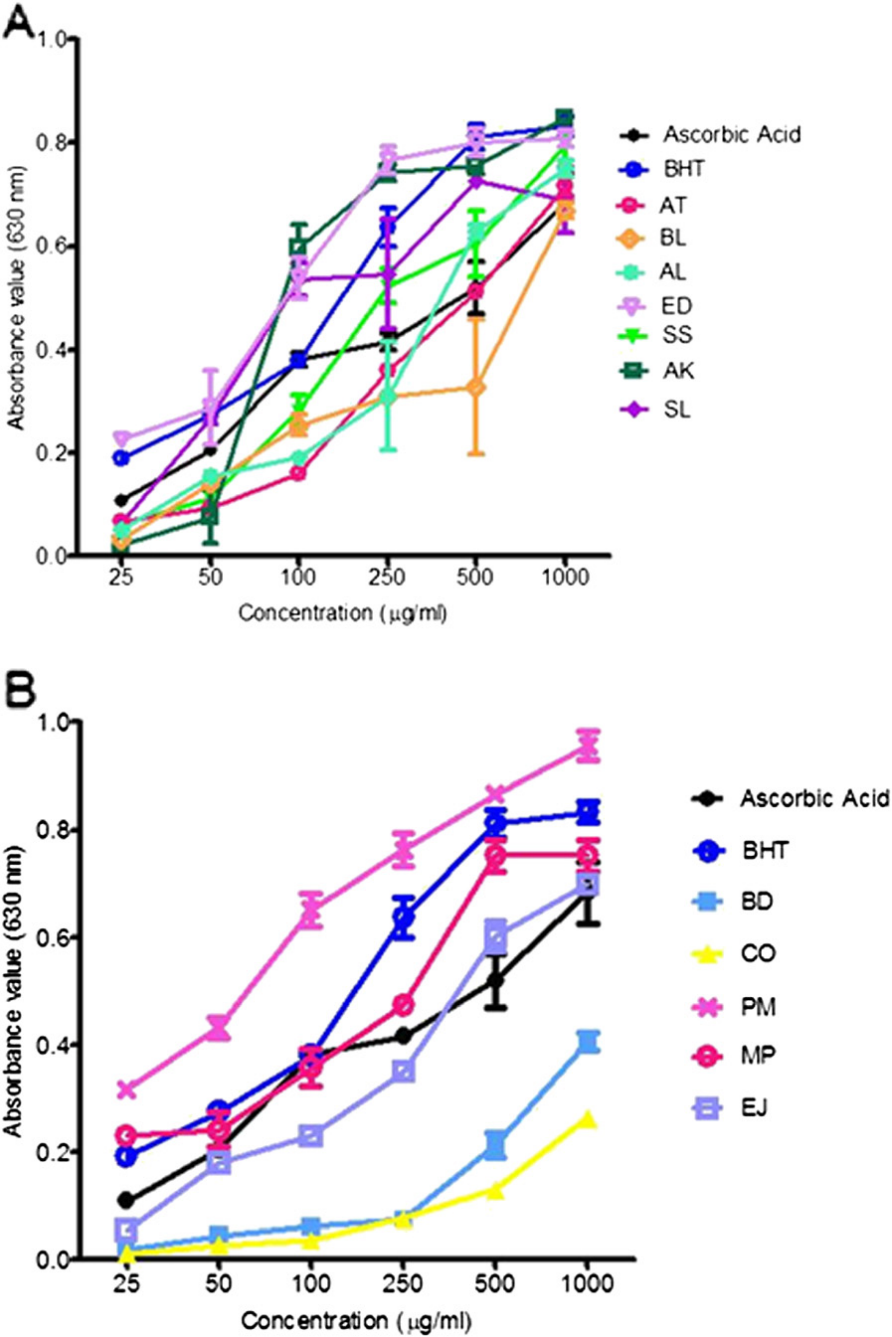
**Ferric ion reducing power activity of selected plants.** Ferric ion-reducing power of extracts of ( **A**) Australian aboriginal plants and ( **B**) Indian Ayurvedic plants ethanolic extracts, showing a dose-dependent linear increase in absorbance. Each value represents the mean ± SEM of triplicates experiments.

Antioxidant activities of these plant extracts were assessed through their ability to reduce the Fe^3+^/ferricyanide complex to the ferrous (Fe^2+^) form. The ferrous ion was monitored by measuring the formation of Perl’s Prussian blue at 630 nm [49]. Figure 5 presents the dose-dependent ferric-reducing powers of the sample extracts, ascorbic acid and BHT. The reducing power of ED extract showed similar activity to BHT but higher than ascorbic acid. Among the Indian plant extracts, PM showed good potential whereas BD and CO showed moderate activity. Overall, it was seen that most of the extracts possessed promising antioxidant activity.

Gallic acid and hydroxycinnamic acids are the phenolic acids found commonly in plants. Plants which contain high levels of phenolics are considered a good source of antioxidants and therefore it is important to quantify the total phenolics and total flavonoids in plant extracts as they might have some beneficial effects on health [50]. The colorimetric assays used here are based on the chemical reduction of a reagent. For the extracts tested in this study, total phenolic content ranged from 0.42 - 30.27 μg/mg gallic acid and total flavonoids ranged from 0.51 - 32.94 μg/mg quercetin equivalents (Table 2). Pearson’s correlation coefficient between total flavonoids and total phenolic was 0.796.

**Table 2:** Total phenolic and flavonoid contents for Australian aboriginal and Indian Ayurvedic plant extracts

** Plant extract**	**TPC (μg GAE/mg extract)**	**TFC (μg QE/mg extract)**
***Santalum spicatum***	0.87 ± 0.11	1.37 ± 0.11
***Acacia ligulata***	0.95 ± 0.06	1.52 ± 0.36
***Euphorbia drummondii***	0.93 ± 0.01	0.51 ± 0.12
***Beyeria leshnaultii***	0.42 ± 0.038	1.34 ± 0.21
***Acacia kempeana***	1.47 ± 0.075	1.78 ± 0.15
***Acacia tetragonophylla***	0.56 ± 0.17	0.94 ± 0.13
***Santalum lanceolatum***	1.28 ± 0.035	1.35 ± 0.18
***Eugenia jambolana***	28.31 ± 0.22	10.72 ± 0.44
***Curculigo orchoides***	15.17 ± 0.42	22.18 ± 0.43
***Pterocarpus marsupium***	30.27 ± 0.88	32.94 ± 3.24
***Boerhaavia diffusa***	5.54 ± 0.24	3.58 ± 0.61
***Mucuna pruriens***	5.83 ± 0.57	13.25 ± 3.7

TPC – total phenolic content, TFC – total flavonoid content.

GAE- gallic acid equivalent, QE- quercetin equivalent.

The results are average of triplicate analysis (n = 3; data expressed as mean ± SD).

Although there are scientific reports about anti-diabetic activities of many Indian plants, there are no such studies on the activity of Australian plants with respect to diabetes. The Australian medicinal plants investigated in this study (SS, AL, ED, BL, AK, AT and SL) have been used for general illness, cold, cough and pain [23]. However, this is the first study to assess the potential of Australian traditional medicinal plants to be used in the management of hyperglycemia and diabetes. The traditional hunter-gatherer lifestyle and diet of Aboriginal people, which was high in carbohydrates, fibre, proteins and nutrients but low in fat and sugars, meant that cardiovascular diseases and diabetes were not common in these people. After the settlement of Europeans, the diet became Westernized with high sugar and fat content and the lack of essential nutrients, vitamins, minerals, proteins and fibre. This has increased the disease risk and these disorders are now prevalent in indigenous populations with the incidence of type 2 diabetes rapidly increasing [51,52].

Flavonoids, alkaloids and triterpenoids may be related to the anti-diabetic activity of plants. In particular, flavonoids are responsible for variety of pharmacological activities. For example, epicatechin is known to possess insulin-like properties, while epigallocatechin gallate is considered a promising hypoglycemic agent [53]. As shown in previous studies, the enzyme inhibition activity may be related to the polyphenolic content of the plant extract, however further studies are needed to confirm this.

## Conclusions

The present study showed the anti-diabetic potential of a number of medicinal plant extracts. Most of the plants showed promise as agents for the management of hyperglycemia, along with good antioxidant activity. Indeed, the antioxidant and α-amylase and α-glucosidase inhibitory activities of plants and foods have been associated with their anti-diabetic activity [42,54,55]. Indian Ayurvedic plants have been previously reported to have anti-diabetic properties; however limited data were available about the mechanism of action. Therefore, the plants should be further investigated as they may provide leads for the discovery of new drugs for the management of diabetes with minimal side effects.

## Abbreviations

WHO: World Health Organisation; DPPH: 2 2-diphenyl-1-picryl hydrazyl; DNJ: 1- deoxynojirimycin; ROS: Reactive Oxygen Species; SD: Standard Deviation.

## Competing interests

The authors declare that they have no competing interests.

## Authors’ contributions

VG performed the experiments, evaluated the results and wrote the manuscript. IH and EP assisted in experimental design, evaluated the results and corrected the manuscript. Authors read and approved the final manuscript.

## Pre-publication history

The pre-publication history for this paper can be accessed here:

http://www.biomedcentral.com/1472-6882/12/77/prepub
